# Effects of Chlortetracycline on Lignin Biosynthesis in *Arabidopsis thaliana*

**DOI:** 10.3390/ijms26052288

**Published:** 2025-03-04

**Authors:** Aaron Newborn, Ayesha Karamat, Benoit Van Aken

**Affiliations:** 1 Department of Chemistry & Biochemistry, George Mason University, Fairfax, VA 22030, USA; anewborn@gmu.edu; 2 Department of Environmental Science & Policy, George Mason University, Fairfax, VA 22030, USA; akaramat@gmu.edu

**Keywords:** *Arabidopsis thaliana*, chlortetracycline, Fourier-transform infrared spectroscopy (FTIR), lignin, RNA sequencing, transcriptomics

## Abstract

Feedstock plants for biofuel production can be cultivated on polluted sites that are unsuitable for edible crop production. This approach combines environmental restoration and renewable energy production, therefore enhancing the economic viability of plant-derived biofuels. Previous studies have indicated that exposure to environmental pollutants may elevate lignin levels in exposed plants, potentially impacting the biomass digestibility and the efficiency of bioethanol conversion. In this study, we investigated the impact of the antimicrobial agent chlortetracycline on lignin biosynthesis in the reference organism *Arabidopsis thaliana*. Toxicity testing showed that exposure to chlortetracycline significantly reduced plant growth at concentrations above 2.5 mg L^−1^. Using Fourier-transform infrared spectroscopy (FTIR) analysis, we observed a significant increase in the lignin signature, ranging from 16 to 40%, in plants exposed to chlortetracycline as compared to non-exposed control plants. Transcriptomic analysis (RNA sequencing) was conducted to determine the molecular basis of plant response to chlortetracycline, revealing significant enrichment of several genes involved in lignin biosynthesis and the phenylpropanoid pathway, including cinnamyl alcohol dehydrogenase and peroxidases. Exposure to chlortetracycline also resulted in the overexpression of genes involved in the metabolism of xenobiotic compounds, including cytochrome P450 monooxygenases, glutathione *S*-transferases, and glycosyltransferases. Chlortetracycline also induced several genes involved in plant response to stress and defense mechanisms, including transcription factors (e.g., WRKY, MYB, AP2/ERF families), pathogenesis-related proteins, and genes involved in stress signaling. These results suggest that the antibiotic chlortetracycline triggers multiple stress responses in *A. thaliana*, which may cause changes in lignin biosynthesis, reductions in plant growth, increases in the lignin content, and induction of defense metabolic pathways.

## 1. Introduction

Lignin represents a significant barrier to the digestion of plant lignocellulosic biomass and its subsequent conversion into bioethanol [1]. Producing bioethanol has been deemed one of the most sustainable options for reducing dependence on petroleum-based fossil fuels, while it can also simultaneously mitigate greenhouse gas emissions [2]. Additionally, plants grown for biofuel production can be used to rehabilitate polluted land that is unsuitable for residential or agricultural use, a process known as phytoremediation [3,4]. The combined use of phytoremediation and production of lignocellulosic feedstock plants for bioenergy production is emerging as a promising strategy, bringing great environmental and economic benefits [5,6]. Higher plants have demonstrated an ability to take up and, in some cases, metabolize a variety of environmental pollutants, such as polychlorinated biphenyls, chlorinated solvents, pesticides, pharmaceuticals, and metals [3,4,7,8]. A few studies have explored the idea of bioremediation through the growth of bioenergy plants. In one such study, Shi and Cai [9] investigated the cadmium (Cd) accumulation and tolerance of eight potential bioenergy production crops, including soybean, hemp, and flax. A similar study by Fässler et al. [5] explored the possibilities of growing maize, sunflower, and tobacco in a crop rotation to remove the heavy metals zinc and cadmium from the soil.

The calorific value of ethanol is approximately 26.7 MJ kg^−1^, which is significant, even though it is lower than that of gasoline (44–46 MJ kg^−1^). Considering its energy content, the cost of lignocellulosic materials is estimated to be 50% lower than other feedstocks, such as crude oil, corn kernels, and soy oil [10]. While the strategy for growing bioenergy crops on contaminated land is conceptually attractive, this approach may be hindered by an increase in the lignin content frequently induced by exposure to toxic contaminants. Lignin surrounds the matrix of the energy-rich compounds cellulose and hemicellulose, and acts as a technical barrier that may prevent the hydrolytic enzymes from accessing fermentable sugar compounds [2,11,12,13]. Consequently, sustained efforts have focused on optimizing biomass processing for bioethanol production, leading to multiple strategies using mechanical, physicochemical, and enzymatic methods [14]. Lignin serves as the plant’s primary defense against both biotic and abiotic stressors, such as diseases, insects, drought, salt, and toxic contaminants [15,16,17,18]. Exposure to environmental contaminants, such as PCBs, heavy metals, and ozone, was shown to cause an increase in the lignin content and/or an alteration of the lignin composition in various plant species [17,19,20,21,22]. In addition, to potentially reduce the biomass digestibility, toxic contaminants may impair enzymatic processes used for bioethanol production. Toxic compounds from chemical or physicochemical pretreatments of lignin (e.g., furan derivatives and phenols) may impair further enzymatic treatments such as the saccharification and fermentation of the hydrolysates [14,23].

The goal of this study was to investigate the effects of the veterinary antibiotic, chlortetracycline, on lignin biosynthesis in the model plant *Arabidopsis thaliana.* As a member of the tetracycline family, chlortetracycline has been widely used since its discovery in 1945 for both human and animal care [24]. Chlortetracycline is utilized in agriculture to prevent disease and increase growth rates in the livestock and poultry industries. Due to its high excretion rate (70%) and long half-life, chlortetracycline is released into the environment through land-applied manure and effluents from concentrated animal feeding operations (CAFOs) [25,26]. Accumulation of chlortetracycline in agricultural soils frequently reaches concentrations ranging from 100 to 1700 μg kg^−1^ [27]. This enables chlortetracycline to be absorbed, taken up, and, to some degree, metabolized by plants [28,29,30].

Chlortetracycline and other members of the tetracycline family have significant effects on plants and the associated rhizosphere microbial communities. These antibiotics inhibit plant growth, with studies reporting reduced root length, decreased chlorophyll content, and impaired photosynthetic efficiency in various plant species [27,29,31]. Studying the effects of chlortetracycline on eggplants, Li et al. [27] showed a concentration-dependent decrease in plant biomass and chlorophyll fluorescence parameters, indicating disrupted photosynthetic processes. A greenhouse study to evaluate the effects of chlortetracycline and oxytetracycline on pinto bean (*Phaseolus vulgaris*) showed that the compounds can cause plant mortality at a concentration of 160 mg L^−1^ [31]. Besides direct effects on plants, tetracyclines can alter the microbial community structure, richness, and diversity in the plant rhizosphere. Tetracyclines can enrich certain bacterial genera involved in nitrogen cycling while reducing others, potentially disrupting nutrient cycles in soil [27,32].

Lignin is a complex aromatic polymer that plays a crucial role in plant structure and function. It provides rigidity, contributes to water transport, and offers resistance against pathogens. The biosynthesis of lignin involves multiple enzymes and genes, including phenylalanine ammonia-lyase, cinnamate-4-hydroxylase, and cinnamyl alcohol dehydrogenases, as well as various peroxidases and laccases [15,16,17,18].

In this study, hydroponic *A. thaliana* plantlets were exposed to toxic levels of chlortetracycline, and the effect on the lignin content was determined using Fourier-transformed infrared (FTIR) spectroscopy. The effect of chlortetracycline was assessed using toxicity testing based on the plant biomass and through transcriptomic analysis using RNA sequencing. FTIR results showed a significant increase in the lignin content in plants exposed to chlortetracycline as compared to non-exposed plants. The transcriptomic analysis indicated the overexpression of genes involved in the phenylpropanoid pathway, response to stress, plant defense, and the metabolism of xenobiotics. These results suggest that chlortetracycline’s toxicity to the *Arabidopsis* plants triggered multiple defense responses, including increased lignification.

## 2. Results and Discussion

### 2.1. Toxicity Testing

**Figure 1:**
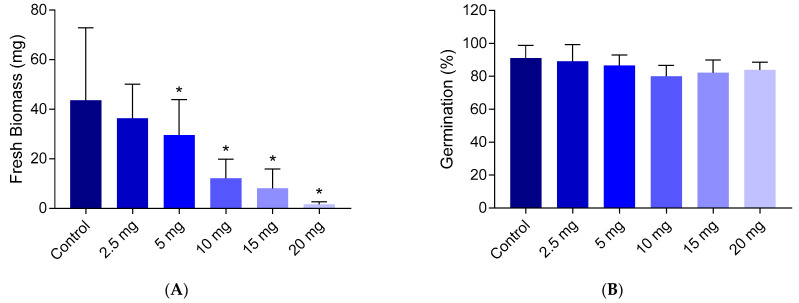
Dose-response of *A. thaliana* exposed to chlortetracycline as determined through the biomass growth after 28 days (panel (**A**)) and germination tests after 7 days (panel (**B**)). The error bars represent standard deviations between biological replicates. The stars indicate statistically significant differences from the control (*t*-test, 95% confidence).

The toxicity of chlortetracycline for *A. thaliana* was determined based on germination and biomass growth tests. Chlortetracycline exhibited a dose-dependent toxicity, with an effect concentration of 50% (EC50) = 8.1 ± 2.3 mg L^−1^, based on biomass growth. The germination rate was not significantly reduced (Figure 1).

While the germination rate showed only minor reductions following exposure to chlortetracycline, the biomass exhibited a marked reduction upon exposure. This is consistent with prior studies, which have indicated that antibiotics like chlortetracycline can significantly hinder plant growth [33,34,35,36,37]. For instance, Cheong et al. [38] observed that while the final germination rate was not affected, chlortetracycline significantly delayed germination in *B. campestris* at concentrations equal to and above 10 mg L^−1^. Furthermore, the subsequent growth phases, where biomass accumulation is critical for plant establishment, were also significantly affected with notably shorter root lengths [38]. These findings further suggest that chlortetracycline impacts not only the initial stages of plant development but also the structural integrity during growth.

### 2.2. Analysis of the Lignin Content by FTIR Spectroscopy

To evaluate how chlortetracycline may affect the biomass composition of *Arabidopsis*, FTIR analyses were conducted on dried, ground whole plantlets. Traditional wet chemistry (e.g., thioacidolysis) and instrumental (e.g., gas chromatography/mass spectrometry (GC/MS)) methods to analyze lignin in plants are time- and labor-intensive, and consume the plant material being investigated [39]. On the contrary, spectroscopy methods (i.e., Raman, near-infrared (NIR), and mid-infrared (MIR)), which are fast, non-destructive, and have potential for high-throughput analysis, have advanced as alternative techniques for the analysis of complex biomasses [40]. Specifically, FTIR has been widely used for the rapid and non-invasive analysis of the lignin content in different plant species [39,40,41,42]. For instance, FTIR using attenuated total reflectance (ATR) for lignin quantification in polar wood samples has been validated by systematic comparison with the acetyl bromide method, returning coefficients of determination R^2^ = 0.91 and 0.81 for calibration and cross-validation, respectively [43]. In a similar study, Javier-Astete et al. [44] analyzed 300 samples of Capirona (*Calycophyllum spruceanum*) and Bolaina (*Guazuma crinita*) using the acid detergent lignin (ADL) method and built a partial least squares (PLS) model from the FTIR spectra. They found a root mean square error of prediction (RMSEP) = 0.48 and 0.81 for Capirona and Bolaina, respectively. Complex organic material, such as plant tissues, contains numerous molecular components leading to significant overlapping of the vibrational energies on the FTIR spectra, therefore preventing the accurate quantification of specific molecular components. Extraction of relevant information from FTIR spectra therefore requires proper preprocessing and multivariate analysis to selectively distinguish specific vibrational energies. In this study, meaningful peaks were resolved by the second-derivative transformation of the FTIR spectra (Figure S1). This approach has been largely used to detect changes in the biomass composition of plants exposed to various stressors, including toxic species [45,46,47,48].

**Figure 2:**
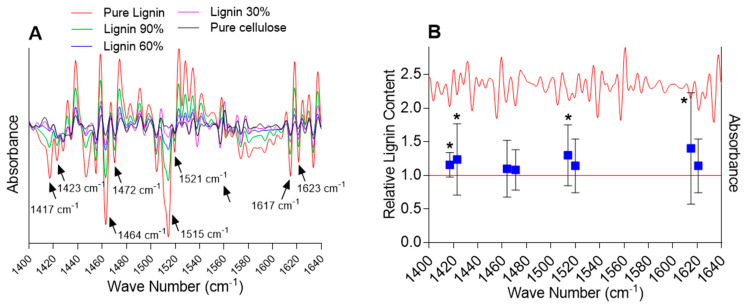
Selected second-derivative FTIR spectra of lignin/cellulose standards (panel (**A**)) and *Arabidopsis* samples exposed to chlortetracycline and non-exposed controls (panel (**B**)). The five peaks used for lignin quantification are shown. The raw spectra were processed with basic ATR correction, unit vector normalization (UVN), Savitzky–Golay second derivative, and extended multiplicative scatter correction (EMSC). The stars indicate statistically significant differences from the control (*t*-test, 95% confidence).

Quantification of the lignin content first required identifying *lignin-specific* peaks in the FTIR spectra. A comparison of the second-derivative transformed spectra of lignin standards with the spectra of *Arabidopsis* samples was performed in the fingerprint region (~800 to 1800 cm^−1^), allowing us to identify 10 distinct lignin-specific peaks in the *Arabidopsis* spectra. Next, standard curves (second-derivative peak height versus lignin content) were drawn, and the peaks showing a significant correlation between peak height and lignin content were further selected. Eight peaks exhibited strong correlations with the lignin content (Pearson’s correlation coefficients r ≥ 0.75, *p*-value < 0.05) and were utilized to estimate the relative change in the biomass composition between the plants exposed to chlortetracycline and the control plants: 1417, 1423, 1464, 1472, 1515, 1521, 1617, and 1623 cm^−1^ (Figure 2).

**Figure 3:**
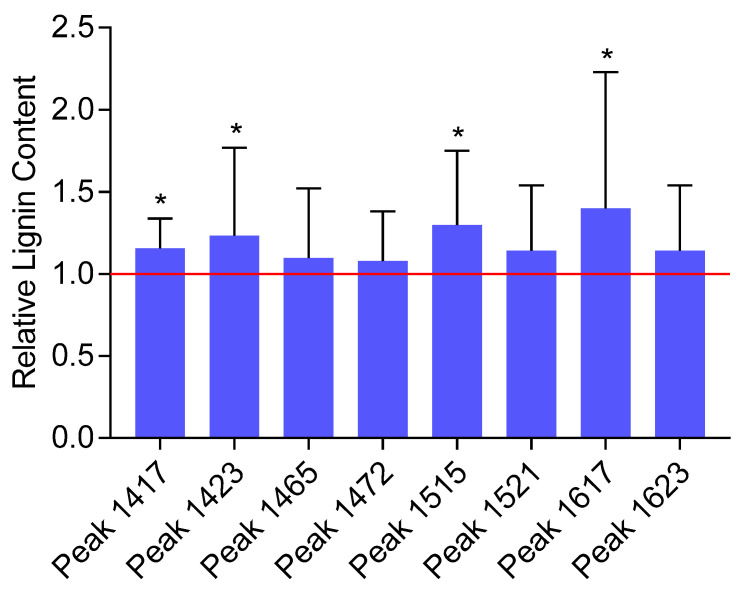
Lignin content in plants exposed to chlortetracycline relative to the control, non-exposed plants as estimated by FTIR second-derivative peaks. The error bars represent the standard deviations. The stars indicate statistically significant differences between exposed and control plants at the 95% confidence level.

While an increase in the mean lignin content upon exposure to chlortetracycline was detected for all the eight selected *lignin-specific* peaks, only four peaks showed a statistically significant change in the lignin content (i.e., 1417, 1423, 1515, and 1617 cm^−1^) ranging from 15.6 to 40.1% (Figure 3). Based on the literature, these four peaks correspond to specific vibrational modes associated with lignin’s aromatic ring stretching and deformation. The 1417 cm^−1^ peak is commonly associated with lignin and represents aromatic ring vibrations and CH deformations [49,50]. The 1423 cm^−1^ peak is also commonly associated with lignin and represents skeletal vibrations of aromatic rings, bending vibrations of O–CH_3_, and scissoring vibrations of CH_2_ [49,51]. The 1515 cm^−1^ peak is indicative of aromatic skeletal vibrations, and the 1617 cm^−1^ peak is associated with the aromatic ring stretching coupled with C=O stretching [49,51,52]. Although it is very unlikely that the very small amount of chlortetracycline taken up by plants would result in a noticeable FTIR signal, spectra of the pure compounds were recorded to ensure that they did not contribute to the lignin-specific peaks (data not presented). FTIR spectra of the pure compound did not contain peaks susceptible to interfering with the significant lignin-specific peaks. Although some variability was observed among biological replicates, the overall FTIR analysis revealed a significant increase in the relative lignin content in samples exposed to chlortetracycline as compared with the non-exposed samples.

Higher plants have evolved multiple physiological and molecular mechanisms to respond to external stress (e.g., drought, salts, heat, and heavy metals). In particular, exposure to stress induces changes in the composition and structure of the cell wall, which constitutes physical and chemical protection against external factors [22]. Our results align with prior studies showing an increase in the lignin content in plants exposed to toxic stress (e.g., metals, PCBs) [15,16,17,18,19,21]. For instance, aluminum toxicity has been shown to increase the lignin content and expression of genes involved in lignin synthesis in different species, including Scots pine (*Pinus sylvestris*), black tea-tree (*Melaleuca bracteata*), *Citrus sinensis,* and *Citrus grandis* [22]. Exposure to cadmium was shown to increase the lignin content in pine trees (*Pinus sylvestris*) by inducing the expression of peroxidases mediating lignin polymerization [53]. Similarly, exposure to Cu and Zn has been related to an increase in the lignin content and genes involved in lignin biosynthesis in several herbaceous plants including *Matricaria chamomilla*, *Oryza sativa*, and *A. thaliana* [22]. To the best of our knowledge, only one study showed that exposure to organic contaminants (PCBs) resulted in increasing lignification in plants [21]. In some instances, exposure to metals may also result in a reduction in the lignin content. Ghanati et al. (2005) reported that exposure of tea (*Camellia sinensis*) to aluminum stress (400 μM) reduced the activities of key enzymes involved in lignin synthesis, leading to a decrease in lignin content, which has been related to enhanced growth of the plants.

Besides affecting the biomass lignin content, abiotic stresses have been shown to alter the composition of lignin, which may also have implications for lignin digestion and its conversion into bioethanol. Lignin is a complex polymer made by random polymerization of three primary phenylpropane units: the *p*-hydroxyphenyl (H), guaiacyl (G), and syringyl (S) units [54,55]. The relative abundance of these units, and especially the S/G ratio, largely determines the structural properties of lignin and the conversion yield into bioethanol. A higher S/G ratio indicates a more linear lignocellulosic structure with less cross-linking, making lignin more amenable to chemical and enzymatic degradation [14,56]. For instance, Hori et al. [57] reported that exposure of *Populus trichocarpa* to drought and salt stress decreased the lignin S/G ratio, which was consistent with the observed downregulation of genes encoding key enzymes for S-lignin biosynthesis. Similarly, Lima et al. [58] observed that G- and S-lignin increased, while H-lignin decreased in coffee (*Coffea arabica*) under heat stress. However, how the lignin composition may be affected by toxic stress has not been reported in the literature.

### 2.3. Gene Expression Analysis

The response of *Arabidopsis* plantlets exposed to chlortetracycline was investigated using transcriptomic analysis (RNA sequencing). The number of reads (i.e., fragments of mRNA) per sample ranged from 23.8 × 10^6^ to 38.2 × 10^6^, with mean quality scores from 38.1 to 38.2 (91–92% of fragments with a number of bases ≥ 30). After filtering out low-quality and non-significant expression results, 564 genes were shown to be differentially expressed by exposure to chlortetracycline (as compared with non-exposed controls, adjusted *p*-value < 0.05), including 480 (85.1%) upregulated genes (fold change > 2.0) and 84 (14.9%) downregulated genes (fold change < 0.5). Cluster analysis of the gene expression levels (i.e., hierarchical clustering) showed good consistency between the six replicates of each treatment (Figure S2).

The analysis of the transcriptome enables the quantification of changes in gene expression levels in response to environmental stressors. RNA sequencing is increasingly recognized as the gold standard in transcriptomics because it offers more accurate quantification of transcripts compared to other methods, such as microarrays and tag-based techniques.

Enrichment of transcripts in different Gene Ontology (GO) categories was interrogated using DAVID and KEEG_PATHWAY. Overall, exposure to chlortetracycline resulted in the enrichment of genes involved in response to stress and defense systems.

**Figure 4:**
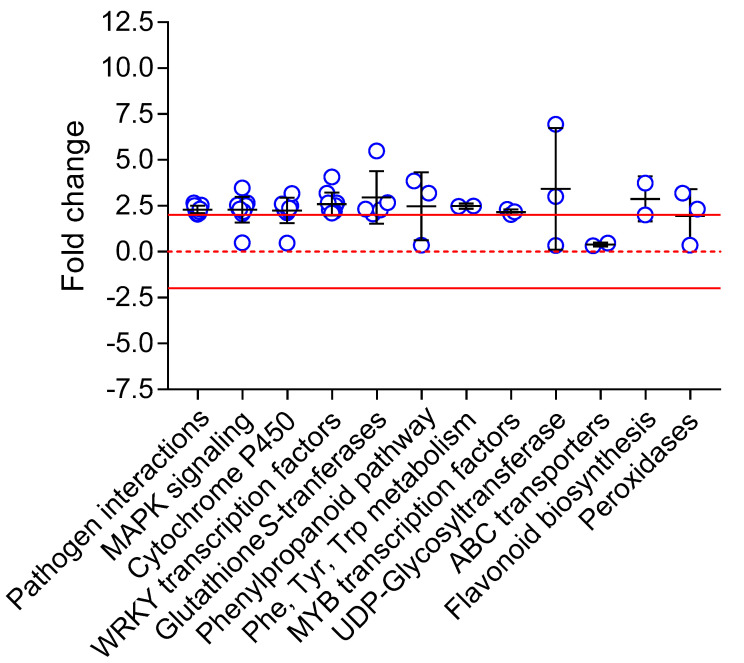
Fold changes of differentially expressed genes (DEGs) in *Arabidopsis* exposed to chlortetracycline. DEGs were grouped based on relevant KEGG objects. The solid red lines represent fold changes of −2 and +2. The red dashed line represents a fold change of zero. Each blue dot represents a specific DEG in its category. The black crosses represent the mean fold change and the standard deviation in each category.

DEGs were searched against the KEGG Pathway database (KEGG Mapper) showing enrichment in genes involved in lignin biosynthesis (phenylpropanoid pathway, phenylalanine/tyrosine/tryptophan biosynthesis), plant defense (pathogen interactions), response to stress (MAPK signaling, WRKY and MYB transcription factors), and xenobiotic metabolism (glutathione *S*-transferase, cytochrome P450 monooxygenases, UDP-glycosyltransferases, ABC transporters, peroxidases) (Figure 4). Alternatively, a gene enrichment analysis based on Biological Function was conducted using the PANTHER Classification System (through TAIR) (Table 1).

This research hypothesized that exposure of plants to toxic compounds, such as the antibiotic chlortetracycline, may increase lignin biosynthesis, therefore potentially impairing biomass conversion for bioethanol production. Lignin biosynthesis includes two major steps: (1) the synthesis of monolignols (i.e., sinapyl alcohol, coniferyl alcohol, and *p*-coumaryl alcohol) through the phenylpropanoid pathway and (2) the polymerization of monolignols through radical coupling [16,17]. We observed that one major enzyme involved in monolignol biosynthesis, cinnamyl alcohol dehydrogenase 8 (AT4G37990, fold change 3.85), was significantly upregulated by exposure to chlortetracycline. Exposure to chlortetracycline also induced expression of a berberine bridge enzyme (BBE)-like enzyme (AT5G44390, fold change 2.04), catalyzing the oxidation of monolignols to their corresponding aldehydes, which, together with monolignols, are building blocks for lignin biosynthesis [59]. These compounds are then polymerized by peroxidases and laccases in the secondary cell wall to form lignin [16,17]. In our study, we observed three upregulated peroxidase genes potentially involved in the phenylpropanoid pathway (AT5G19880, fold change 3.19, and AT4G31870, fold change 2.3). In addition, exposure to the compound induced several genes involved in the biosynthesis of the phenylpropanoid precursors phenylalanine, tyrosine, and tryptophan (AT3G44300, fold change 2.50, and AT2G20340, fold change 2.47).

Evidence from the literature indicates that plant exposure to biotic and abiotic stresses, such as drought, cold, salt, pathogens, and toxic contaminants, triggers cell wall thickening by lignin deposition [17]. Increased lignification enhances the strength of the cell wall, which is an efficient barrier against insects, pathogens, and chemicals. Indeed, the induction of enzymes involved in lignin biosynthesis has been reported in plants exposed to a range of toxic species, including heavy metals, atmospheric ozone, and polychlorinated biphenyls (PCBs) [16,17,19,20,21,60]. In a prior study, our group reported an increase in the lignin content in *Arabidopsis* plants exposed to PCBs, which was associated with the overexpression of genes involved in the phenylpropanoid pathway (e.g., caffeoyl-CoA *O*-methyltransferase, cinnamyl alcohol dehydrogenase), lignin synthesis (cell wall peroxidases), and the biosynthesis of phenylpropanoid precursors. Li et al. [19] reported that exposure of Ginseng (*Panax ginseng*) root cultures to copper increased the activities of several enzymes involved in the phenylpropanoid pathway (e.g., phenylalanine ammonia lyase, cinnamyl alcohol dehydrogenase) and lignin synthesis (e.g., cell wall peroxidases). Jiang and Yan [60] reported that poplar trees (*Populus alba berolinensis*) grown in soil contaminated by lead showed an increase in the lignin content and enhanced activity of polyphenoloxidases and phenylalanine ammonia lyases. According to several authors, increased lignification of the secondary cell wall in response to heavy metals would serve the purpose of preventing cell extension, therefore protecting the plant against further toxic effects of metals [17,19,20]. Similarly, the atmospheric pollutant ozone (O_3_) has been shown to increase the expression of enzymes involved in lignin biosynthesis (phenylalanine ammonia lyase) in different species, including *Arabidopsis*, tobacco, and blueberry [61,62].

Besides the genes involved in lignin biosynthesis, exposure to chlortetracycline also induced genes involved in pathogen response (e.g., plant–pathogen interactions, systemic immune response) as well as in response to various abiotic stresses (e.g., iron starvation, decreased oxygen levels, salt stress, reactive oxygen species) (Figure 4, Table 1). However, no indices of pathogen infection or abiotic stress in our non-exposed plants were detected during the experiments. These could be explained by the fact that plant exposure to one type of stress frequently triggers responses to other types of stressors. It is well recognized that different types of stresses produce overlapping responses at the physiological, hormonal, and transcriptional levels [63,64,65,66,67,68]. For instance, a transcriptomic analysis of *Arabidopsis* exposed to nine different abiotic stresses revealed that 67 genes were upregulated by all stress types [69]. It has been suggested that different types of stresses (e.g., pathogens and toxic species) may induce similar cellular damages (for instance, through oxidative stress and ROS), therefore triggering a similar cellular response [64,66].

Another gene category significantly induced in response to chlortetracycline included genes involved in the xenobiotic metabolism, including glutathione *S*-transferases, glycosyltransferases, cytochrome P-450 monooxygenases, ACB transporter proteins, etc. Although evidence of chlortetracycline metabolism in plants has not been reported, a wide range of organic pollutants have been shown to be, to various extents, enzymatically transformed in plants. The plant metabolism of xenobiotic compounds is conceptually represented as a three-phase process known as the *green liver model* [70]: Phase I, the initial activation, consists of oxidation or reduction of the toxic compound, which confers higher solubility and reactivity. Phase II involves the conjugation of phase I-activated compounds with molecules of plant origin (e.g., glutathione, amino acids, sugars), forming adducts that are less toxic and more soluble than the parent compound. Phase III involves sequestration of the conjugates in plant organelles (e.g., vacuole) or incorporation into plant structures (e.g., cell wall) [7]. In our study, exposure of *Arabidopsis* to chlortetracycline induced a variety of genes potentially involved in the metabolism of xenobiotics, including cytochrome P450 monooxygenases (e.g., AT2G45570, fold change 3.18), glutathione *S*-transferases (e.g., AT3G25180, fold change 2.85), UDP-glycosyltransferases (e.g., AT2G22590, fold change 6.94), and peroxidases (AT4G31870, fold change 2.33). In particular, AT2G29450 and AT2G29470 are members of the TAU glutathione *S*-transferase gene family, which are known to be involved in phase II detoxification processes and are induced by exposure to herbicides [71]. These observations may suggest that *Arabidopsis* is capable of transforming the antibiotic chlortetracycline.

Exposure to chlortetracycline also resulted in the overexpression of many genes encoding transcription factors involved in stress response and potentially in xenobiotic metabolism. Several of these genes are part of the MAPK signaling pathway, which is involved in signal transduction during stress responses. For instance, AT5G59220 (fold change 2.20) encodes a protein phosphatase 2C family member, which is often associated with stress signaling [72]. Other genes encode AP2/ERF, WRKY, and MYB transcription factors: AT1G01250 (fold change 2.83) and AT4G31800 (fold change 4.08) belong to the AP2/ERF transcription factor family, which induces responses to various abiotic stresses, including xenobiotic stress [73]. AT2G38470 (fold change 2.51) and AT4G23810 (fold change 2.20) encode WRKY transcription factors that are known to play roles in both biotic and abiotic stress responses, potentially including xenobiotic stress [74]. AT5G67300 (fold change 2.17) encodes MYB44, a member of the MYB transcription factor family, which is involved in various stress responses [75].

## 3. Experimental Section

### 3.1. Chemicals

Chlortetracycline hydrochloride was purchased in ≥99% purity from Thermo Fisher (Allentown, PA, USA). Lignin (low-sulfate kraft lignin) and cellulose standards (from cotton linters) were obtained from Sigma-Aldrich (St. Louis, MO, USA). Murashige and Skoog medium was obtained from Caisson Labs (Smithfield, UT, USA). Phytoagar was obtained from Plant Media (Dublin, OH, USA). Other reagents and solvents were obtained from Thermo Fisher or Sigma-Aldrich in analytical purity.

### 3.2. Toxicity Tests

*A. thaliana*, ecotype Columbia (Col-0/Redei-L211497, Ohio State University, Columbus, OH, USA), was grown as we described in [21,76] with some modifications. In short, plants were grown under sterile conditions in Magenta boxes filled with semi-solid medium containing 4.31 g L^−1^ Murashige and Skoog (MS) medium with 3% sucrose, 0.5 g L^−1^ 2-(*N*-morpholino)ethanesulfonic acid (MES), and 6.0 g L^−1^ Phytoagar [77]. The pH was adjusted to 5.7 and the medium was sterilized by autoclave. For exposure to chlortetracycline, the medium was supplemented with the compound formulated in methanol stock solutions (2500 mg L^−1^) at concentrations ranging from 0 to 25 mg L^−1^, while control media were supplemented with an equivalent amount of solvent carrier (methanol) only. These environmentally relevant concentrations have previously been shown to cause mild toxic effects on plants [35,37]. Seeds were surface-sterilized by immersion in 5% commercial bleach containing one drop of Tween^®^ 20 for 10 min, then vernalized in the dark at 4 °C for 3 days [77]. Seeds were then placed in the Magenta boxes (9 seeds per box) and incubated at 25 °C under white (cool) fluorescent light (0.4 ± 0.05 W ft^−2^) with a 16 h light/8 h dark photoperiod. After 7 days, the germination rate was determined by visual observation. After 28 days, the plantlets were removed from the medium, washed, and weighed to determine the fresh biomass.

### 3.3. FTIR Analysis

Plant samples were oven-dried at 60 °C for 48 h and then ground to a fine powder in a centrifuge tube using a disposable pestle. A total of 49 plant samples were analyzed, which included 16 plants exposed to chlortetracycline and 33 non-exposed control plants. Additionally, 13 lignin (low-sulfate kraft lignin) and cellulose (from cotton linters) binary mixtures were generated by first oven-drying pure powdered standards at 60 °C for 48 h and then mixing the two components in defined weight ratios ranging from pure lignin to pure cellulose. Homogenization was achieved by shaking the powder mix in 4 mL glass vials at 2500 rpm for 30 min.

FTIR spectra were collected from approximately 200 mg of sample or standard using a Nicolet iS20 FTIR spectrometer equipped with a DTGS KBr detector and a Smart iTX diamond attenuated total reflection (ATR) device (Thermo Fisher): number of scans = 32, resolution = 4.0 cm^−1^, and gain = 4. Spectra were collected in the 4000–400 cm^−1^ region using OMNIC version 9 (Thermo Fisher). Three spectra were collected for each standard and sample, which were averaged for the subsequent processing.

Pretreatment and statistical analyses of the FTIR data were performed with Aspen Unscrambler version 14.2 (Aspen Technology, Bedford, MA, USA), Microsoft Excel, and SPSS version 26.0 (IBM, Chicago, IL, USA). In brief, spectra were subjected to basic ATR correction, normalization using unit vector normalization (UVN), Savitzky–Golay second-derivative filtering (15-point smoothing, 2nd polynomial order), and extended multiplicative scatter correction (EMSC) [39]. Comparison of the means of second-derivative peak heights was performed on SPSS using two-tailed *t*-tests. The equality of the variance was determined using Levene’s test. For non-normal distributions (based on Kolmogorov–Smirnov and Shapiro–Wilk normality tests), the nonparametric Kruskal–Wallis test was used.

### 3.4. RNA Sequencing

Immediately after harvest, plants were treated with RNA*later* (Ambion, Austin, TX, USA) and stored at −80 °C. Total RNA was extracted from the whole plants using the TRIzol^®^ Plus RNA Purification kit with on-column PureLink^®^ DNase treatment (Thermo Fisher) as we described in [21]. RNA quality was assessed by the optical density ratios OD_260_/OD_280_ and OD_260_/OD_230_ (NanoDrop™ One*C*, Life Technologies, Frederick, MD, USA) and RNA integrity numbers (RINs) (Tapestation, Agilent, Santa Clara, CA, USA). Twelve RNA samples were selected (six per treatment) and sequencing was performed by GENEWIZ (South Plainfield, NJ, USA) using the standard protocol for total RNA analysis. RNA sequencing libraries were prepared using the NEBNext Ultra RNA Library Prep Kit for Illumina following the manufacturer’s instructions (NEB, Ipswich, MA, USA). The sequencing libraries were validated on an Agilent TapeStation (Agilent), and quantified by using a Qubit 2.0 Fluorometer (Invitrogen, Carlsbad, CA, USA) and by qPCR. The libraries were sequenced using an Illumina HiSeq 4000 instrument (San Diego, CA, USA) using a 2 × 150 bp Paired-End (PE) configuration. Image analysis and base calling were conducted by HiSeq Control Software (HCS). Raw sequence data (.bcl files) generated from the Illumina HiSeq were converted into fastq files and de-multiplexed using Illumina’s bcl2fastq version 2.17 software. One mismatch was allowed for index sequence identification.

### 3.5. Sequencing Data Analysis

Differential expression analysis was performed using the DESeq2 package (R version 4.0/Bioconductor version 3.12) with a fold change ≥ 2 and a false discovery rate (FDR)-adjusted *p*-value (Benjamini and Hochberg) < 0.05 [78]. Differentially expressed genes (DEGs) involved in lignin biosynthesis and other relevant functional categories were searched against the KEGG PATHWAY database (www.genome.jp/kegg/, accessed on 3 March 2025). DEGs were grouped based on relevant KEGG objects (i.e., pathways, BRITE functional hierarchy, and KEGG modules). Enrichment of genes in different Gene Ontology (GO) categories was interrogated using DAVID (https://davidbioinformatics.nih.gov/, accessed on 3 March 2025) version 6.8 using Functional Annotation Clustering [79,80] and TAIR (The Arabidopsis Information Resource, www.arabidopsis.org/, accessed on 3 March 2025) using the PANTHER Classification System (www.pantherdb.org/, accessed on 3 March 2025). Only terms with positive enrichment ≥ 1.5 and FDR-adjusted *p*-value (Bonferroni) < 0.05 were considered for further discussion.

### 3.6. Statistical Analyses

The IC_50_ value for biomass was determined after performing a curve fitting of the toxicity data using the Inhibitor vs. Response—Variable Slope (four parameters) model (Prism version 10.4.1, GraphPad, Boston, MA, USA). The statistical significance of the differences between control and treatment groups was assessed using unpaired *t*-tests. The homoscedasticity of the data was tested using the nonparametric correlation test (GraphPad Prism version 10.4.1). When the assumption of homoscedasticity was not met, the unpaired *t*-test with Welch’s correction was used (GraphPad Prism version 10.4.1).

## 4. Conclusions

The 2007 U.S. Energy Independence & Security Act aims to significantly increase the production of renewable fuel, largely from bioenergy. However, this creates competition between biofuel crops and food production. Lignocellulosic plants like switchgrass offer a solution, as they can grow on contaminated land, supporting bioremediation and enhancing biodiversity without compromising food security.

Growing bioenergy plants on contaminated land presents an attractive solution that aligns with circular economy principles while offering substantial economic and environmental benefits. This approach transforms unproductive, polluted areas into valuable assets for renewable energy production, not only generating clean energy but also creating jobs in rural areas, supporting local economies [81]. From an environmental perspective, these bioenergy systems can deliver significant greenhouse gas savings compared to fossil fuels. Moreover, perennial energy crops can improve soil quality over time, potentially restoring degraded lands [82]. Combining bioenergy production with phytoremediation offers a dual benefit of energy generation and land restoration, effectively closing the loop on resource use while addressing environmental challenges [23].

While exposure to inorganic contaminants, such as heavy metals and ozone, was shown to cause an increase in the lignin content in various plant species, there is limited knowledge regarding the impact of organic compounds on lignin biosynthesis in plants. Only one prior publication reports that PCBs increased lignification in exposed Arabidopsis plants [21]. The present study showed for the first time that exposure to chlortetracycline, which is widespread in contaminated agricultural soils, induces genes involved in lignin biosynthesis and increases the lignin content in *Arabidopsis* plants, which has potential implications for the production of bioethanol. Understanding the molecular mechanisms of lignin biosynthesis under toxic stress could help mitigate these effects.

Lignin’s biodegradability and renewability make it a promising raw material for various products. Studying how environmental stressors affect lignin formation could advance the development of industrially valuable lignin properties.

## Figures and Tables

**Table 1:** Enrichment analysis using Panther. Only enrichment categories relevant to lignin biosynthesis and toxic stress response are shown (a full version of the table is presented in Supplemental Information: Table S1). Fold enrichment values are given together with the Bonferroni-adjusted *p*-value. Only enrichment terms with Bonferroni-adjusted *p*-value < 0.05 are shown.

GO Biological Process Term	Total Genes	# DEGs	# DEGs Expected	Fold Enrichment	Bonferroni *p*-Value
Cellular response to sulfur starvation (GO:0010438)	7	4	0.13	29.68	1.25 × 10^−2^
Cellular response to iron ion starvation (GO:0010106)	8	4	0.15	25.97	2.46 × 10^−2^
Priming of cellular response to stress (GO:0080136)	13	6	0.25	23.97	2.08 × 10^−4^
Systemic acquired resistance (GO:0009627)	67	13	1.29	10.08	1.17× 10^−6^
Cellular response to decreased oxygen levels (GO:0036294)	239	28	4.6	6.08	7.25 × 10^−11^
Innate immune response (GO:0045087)	183	16	3.52	4.54	1.57 × 10^−3^
Response to reactive oxygen species (GO:0000302)	166	14	3.2	4.38	1.21 × 10^−2^
Defense response to bacterium (GO:0042742)	389	31	7.49	4.14	8.96 × 10^−8^
Regulation of response to stress (GO:0080134)	474	31	9.13	3.4	1.13 × 10^−5^
Response to external stimulus (GO:0009605)	1410	83	27.15	3.06	2.07 × 10^−16^
Response to osmotic stress (GO:0006970)	579	34	11.15	3.05	3.01 × 10^−5^
Response to salt stress (GO:0009651)	482	28	9.28	3.02	7.52 × 10^−4^
Cellular response to chemical stimulus (GO:0070887)	1133	62	21.81	2.84	4.19 × 10^−10^
Response to chemical (GO:0042221)	2743	134	52.81	2.54	4.21 × 10^−21^
Response to abiotic stimulus (GO:0009628)	2257	93	43.46	2.14	5.86 × 10^−9^
Macromolecule biosynthetic process (GO:0009059)	2351	13	45.27	0.29	2.69 × 10^−5^
Gene expression (GO:0010467)	1924	8	37.04	0.22	2.01 × 10^−5^
Nucleic acid metabolic process (GO:0090304)	1870	7	36	0.19	7.49 × 10^−6^

## Data Availability

The datasets presented in this article are not readily available because data are part of ongoing studies. Requests to access the datasets should be directed to Benoit Van Aken.

## Supplementary Materials

The following supporting information can be downloaded at https://www.mdpi.com/article/10.3390/ijms26052288/s1.

## Abbreviations

FTIR	Fourier-transform infrared spectroscopy
ATR	Attenuated total reflection
PCB	Polychlorinated biphenyl
UVN	Unit vector normalization
EMSC	Extended multiplicative scatter correction
FDR	False discovery rate
DEG	Differentially expressed gene
TAIR	The Arabidopsis Information Resource
GO	Gene Ontology
KEGG	Kyoto Encyclopedia of Genes and Genomes
