# Supplementary material for: Effects of Chlortetracycline on Lignin Biosynthesis in Arabidopsis thaliana
Source: Int J Mol Sci. 2025 Mar 4;26(5):2288. doi: 10.3390/ijms26052288 (PMC11899738; doi:10.3390/ijms26052288)
Supplement: Supplementary file 1 [file ijms-26-02288-s001.zip › ijms-3458782-supplementary.pdf]

Effects of Chlortetracycline on Lignin Biosynthesis in *Arabidopsis thaliana*

Aaron Newborn[1], Ayesha Karamat[2] and Benoit Van Aken[2]*

[1]Department of Chemistry & Biochemistry, George Mason University, Fairfax, Virginia, United States

[2]Department of Environmental Science & Policy, George Mason University, Fairfax, Virginia, United States

*Correspondence: bvanaken@gmu.edu

Supporting Information

Tables

Table S1. Enrichment analysis using Panther. Fold enrichment values are given together with the Bonferroni-adjusted *p*-value. Only enrichment terms with Bonferroni-adjusted *p*-value < 0.05 are shown.

Go biological process term	Total # Genes	# DEGs	# DEGs expected	Fold Enrichment	Bonferroni P-Value
Sulfate assimilation, sulfate reduction by phosphoadenylyl-sulfate reductase (thioredoxin) (GO:0019379)	3	3	0.06	51.94	1.95E-02
Cellular response to sulfur starvation (GO:0010438)	7	4	0.13	29.68	1.25E-02
Cellular response to iron ion starvation (GO:0010106)	8	4	0.15	25.97	2.46E-02
Priming of cellular response to stress (GO:0080136)	13	6	0.25	23.97	2.08E-04
Systemic acquired resistance (GO:0009627)	67	13	1.29	10.08	1.17E-06
Cellular response to hypoxia (GO:0071456)	237	28	4.56	6.14	5.85E-11
Cellular response to decreased oxygen levels (GO:0036294)	239	28	4.6	6.08	7.25E-11
Cellular response to oxygen levels (GO:0071453)	240	28	4.62	6.06	8.06E-11
Response to hypoxia (GO:0001666)	267	30	5.14	5.84	2.65E-11
Response to decreased oxygen levels (GO:0036293)	271	30	5.22	5.75	3.96E-11
Response to oxygen levels (GO:0070482)	273	30	5.26	5.71	4.82E-11
Defense response to symbiont (GO:0140546)	281	30	5.41	5.54	1.04E-10
Innate immune response (GO:0045087)	183	16	3.52	4.54	1.57E-03
Immune response (GO:0006955)	233	20	4.49	4.46	8.41E-05

Response to reactive oxygen species (GO:0000302)	166	14	3.2	4.38	1.21E-02
Response to salicylic acid (GO:0009751)	180	15	3.47	4.33	6.45E-03
Immune system process (GO:0002376)	273	22	5.26	4.19	5.43E-05
Defense response to bacterium (GO:0042742)	389	31	7.49	4.14	8.96E-08
Response to bacterium (GO:0009617)	510	40	9.82	4.07	1.92E-10
Cellular catabolic process (GO:0044248)	247	18	4.76	3.78	4.55E-03
Response to organic cyclic compound (GO:0014070)	331	23	6.37	3.61	3.95E-04
Response to nutrient levels (GO:0031667)	274	19	5.28	3.6	4.93E-03
Defense response to other organism (GO:0098542)	867	60	16.69	3.59	2.87E-14
Defense response (GO:0006952)	1114	77	21.45	3.59	4.19E-19
Regulation of response to stress (GO:0080134)	474	31	9.13	3.4	1.13E-05
Response to external biotic stimulus (GO:0043207)	1197	78	23.05	3.38	7.80E-18
Response to other organism (GO:0051707)	1197	78	23.05	3.38	7.80E-18
Response to biotic stimulus (GO:0009607)	1200	78	23.1	3.38	9.07E-18
Biological process involved in interspecies interaction between organisms (GO:0044419)	1211	78	23.32	3.35	1.57E-17
Response to oxidative stress (GO:0006979)	460	29	8.86	3.27	8.04E-05
Regulation of defense response (GO:0031347)	323	20	6.22	3.22	1.49E-02
Response to fungus (GO:0009620)	386	23	7.43	3.09	5.71E-03
Response to external stimulus (GO:0009605)	1410	83	27.15	3.06	2.07E-16
Response to osmotic stress (GO:0006970)	579	34	11.15	3.05	3.01E-05
Response to salt stress (GO:0009651)	482	28	9.28	3.02	7.52E-04
Response to acid chemical (GO:0001101)	469	27	9.03	2.99	1.46E-03
Cellular response to chemical stimulus (GO:0070887)	1133	62	21.81	2.84	4.19E-10
Response to oxygen-containing compound (GO:1901700)	1593	87	30.67	2.84	2.70E-15
Cellular response to stress (GO:0033554)	1241	66	23.89	2.76	2.38E-10
Response to water (GO:0009415)	436	23	8.39	2.74	4.18E-02
Response to stress (GO:0006950)	3489	172	67.18	2.56	1.11E-29
Response to alcohol (GO:0097305)	549	27	10.57	2.55	2.77E-02
Response to chemical (GO:0042221)	2743	134	52.81	2.54	4.21E-21
Response to lipid (GO:0033993)	906	44	17.44	2.52	6.36E-05
Cellular response to stimulus (GO:0051716)	2877	127	55.39	2.29	4.96E-16
Regulation of response to stimulus (GO:0048583)	939	39	18.08	2.16	2.34E-02
Signaling (GO:0023052)	1665	69	32.06	2.15	5.09E-06
Signal transduction (GO:0007165)	1621	67	31.21	2.15	1.02E-05
Response to abiotic stimulus (GO:0009628)	2257	93	43.46	2.14	5.86E-09

Response to stimulus (GO:0050896)	6004	243	115.6	2.1	9.64E-32
Cell communication (GO:0007154)	1694	68	32.62	2.08	2.41E-05
Response to hormone (GO:0009725)	1394	53	26.84	1.97	7.88E-03
Response to endogenous stimulus (GO:0009719)	1395	53	26.86	1.97	7.96E-03
Biological regulation (GO:0065007)	5900	159	113.6	1.4	8.29E-03
Regulation of biological process (GO:0050789)	5608	151	107.98	1.4	2.00E-02
Unclassified (UNCLASSIFIED)	5909	126	113.77	1.11	0.00E00
Macromolecule metabolic process (GO:0043170)	4742	44	91.3	0.48	9.12E-06
Macromolecule biosynthetic process (GO:0009059)	2351	13	45.27	0.29	2.69E-05
RNA metabolic process (GO:0016070)	1437	7	27.67	0.25	1.12E-02
Gene expression (GO:0010467)	1924	8	37.04	0.22	2.01E-05
Nucleic acid metabolic process (GO:0090304)	1870	7	36	0.19	7.49E-06
Nucleobase-containing compound metabolic process (GO:0006139)	2287	7	44.03	0.16	4.57E-09
Nucleobase-containing compound biosynthetic process (GO:0034654)	1329	4	25.59	0.16	4.82E-04
RNA biosynthetic process (GO:0032774)	1089	3	20.97	0.14	4.09E-03
Nucleic acid biosynthetic process (GO:0141187)	1116	3	21.49	0.14	2.88E-03
RNA processing (GO:0006396)	828	1	15.94	0.06	6.27E-03

Figures

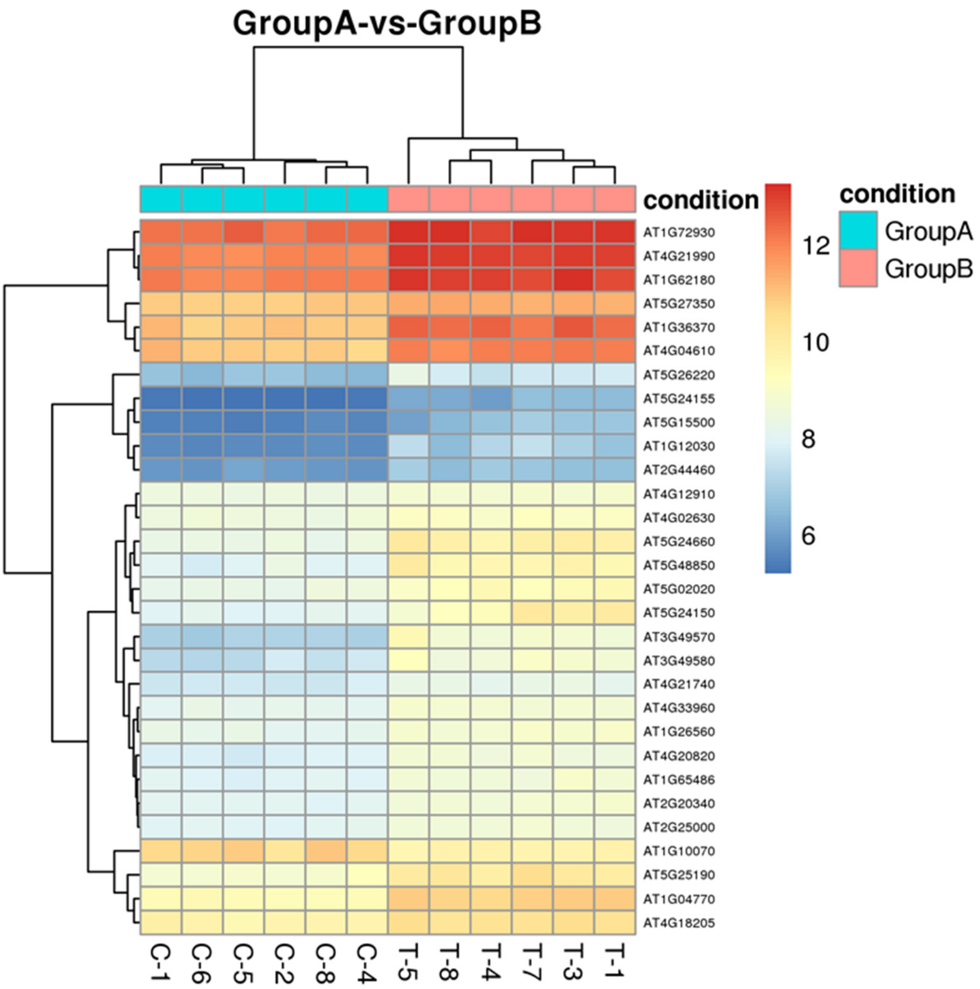

Figure S1. Heatmap showing the hierarchical clustering of gene expression data of the 6 samples exposed to chlortetracycline (C series) and the 6 non-exposed control plants (C series).

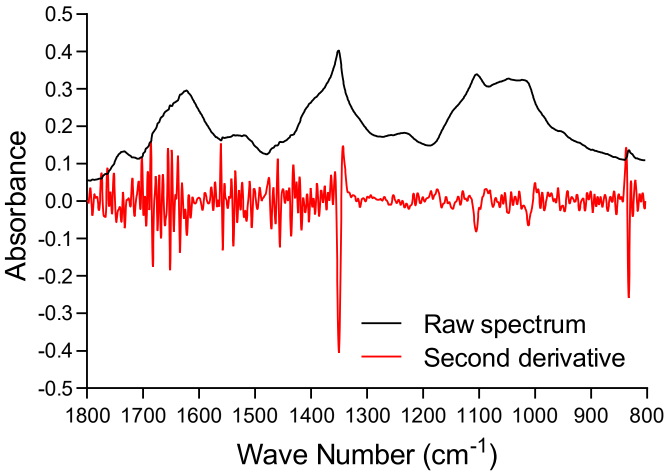

Figure S2. Example of raw and second derivative spectra of an *Arabidopsis* sample (non-exposed control) in the fingerprint region (1800 to 800 cm^{-1}). The raw spectra were processed with basic ATR correction, Savitzky-Golay second derivative filtering (15-point smooth, 2nd polynomial order), and extended multiplicative scatter correction (EMSC).
